# Analysis of CGF Biomolecules, Structure and Cell Population: Characterization of the Stemness Features of CGF Cells and Osteogenic Potential

**DOI:** 10.3390/ijms22168867

**Published:** 2021-08-18

**Authors:** Eleonora Stanca, Nadia Calabriso, Laura Giannotti, Paola Nitti, Fabrizio Damiano, Benedetta Di Chiara Stanca, Maria Annunziata Carluccio, Giuseppe Egidio De Benedetto, Christian Demitri, Andrea Palermo, Franco Ferrante, Luisa Siculella, Alessio Rochira

**Affiliations:** 1 Laboratory of Molecular Biology, Department of Biological and Environmental Sciences and Technologies, University of Salento, 73100 Lecce, Italy; laura.giannotti@unisalento.it (L.G.); fabrizio.damiano@unisalento.it (F.D.); benedetta.dichiara@unisalento.it (B.D.C.S.); 2 National Research Council (CNR) Institute of Clinical Physiology (IFC), 73100 Lecce, Italy; nadia.calabriso@ifc.cnr.it (N.C.); maria.carluccio@ifc.cnr.it (M.A.C.); 3 Department of Engineering for Innovation, Campus Ecotekne, University of Salento, Via per Monteroni, 73100 Lecce, Italy; paola.nitti@unisalento.it (P.N.); christian.demitri@unisalento.it (C.D.); 4 Analytical and Isotopic Mass Spectrometry Laboratory, Department of Cultural Heritage, University of Salento, 73100 Lecce, Italy; giuseppe.debenedetto@unisalento.it; 5 Associate Professor in Implant Dentistry College of Medicine and Dentistry, Birmingham B4 6BN, UK; andrea.palermo2004@libero.it; 6 Specialist in Oral Surgery, Private Practioner, 73100 Lecce, Italy; franco_ferrante@yahoo.it

**Keywords:** CGF, growth factor, stem cells, blood-derived biomaterials, osteogenic differentiation

## Abstract

Concentrated Growth Factors (CGF) represent new autologous (blood-derived biomaterial), attracting growing interest in the field of regenerative medicine. In this study, the chemical, structural, and biological characterization of CGF was carried out. CGF molecular characterization was performed by GC/MS to quantify small metabolites and by ELISA to measure growth factors and matrix metalloproteinases (MMPs) release; structural CGF characterization was carried out by SEM analysis and immunohistochemistry; CGF has been cultured, and its primary cells were isolated for the identification of their surface markers by flow cytometry, Western blot, and real-time PCR; finally, the osteogenic differentiation of CGF primary cells was evaluated through matrix mineralization by alizarin red staining and through mRNA quantification of osteogenic differentiation markers by real-time PCR. We found that CGF has a complex inner structure capable of influencing the release of growth factors, metabolites, and cells. These cells, which could regulate the production and release of the CGF growth factors, show stem features and are able to differentiate into osteoblasts producing a mineralized matrix. These data, taken together, highlight interesting new perspectives for the use of CGF in regenerative medicine.

## 1. Introduction

In the field of regenerative medicine, there is growing interest in platelet concentrates derived from whole blood in order to improve tissue regeneration processes. 

These preparations contain high concentrations of growth factors, such as platelet-derived growth factor (PDGF), transforming growth factors β1 (TGF-β1) and β2 (TGF-β2), vascular endothelial growth factor (VEGF), fibroblast growth factor (FGF), and insulin-like growth factor (IGF), which are all involved in cell proliferation, matrix remodeling and angiogenesis [1]. 

Platelet derivatives have several medical applications, including stimulation of tissue regeneration in dentistry, implantology and plastic surgery, healing of recalcitrant ulcers and burns, repair of musculoskeletal tissue, tendon and ligament lesions, and osteoarthritis treatment [2]. The versatility of these blood derivatives is linked to their autologous nature and simple collection and preparation methods [2]. 

Platelet derivatives can be classified into three different generations based on their characteristics and preparation methods. 

The first generation, developed in the 1970s, is platelet-rich plasma (PRP). It contains several growth factors implicated in tissue repair, but for the fibrin polymerization induction, the preparation requires the use of anticoagulants and bovine thrombin, which interfere with the natural healing process [3,4]. 

The second generation consists of platelet-rich fibrin (PRF). For its preparation, blood samples are collected without using anticoagulants or biological agents. PRF has been further modified into an advanced form called advanced platelet-rich fibrin (A-PRF), which has a fibrin clot softer than PRF and a number of platelet cells greater than PRF [5].

The third and latest generation of platelet derivatives developed by Sacco in 2006 [6] is called concentrated growth factors (CGF), and it can be considered another modified form of PRF. CGF is produced by centrifugation of the blood sample using alternating speed rates. This process leads to a dense fibrin matrix, which can promote the migration of cells, such as fibroblast and endothelial cells [1], and contains more growth factors than PRP and PRF [5,7]. Furthermore, the presence of CD34-positive stem cells, in addition to leukocytes, has been demonstrated in CGF [1]. 

Differences in the growth factors released among PRP, PRF, A-PRF, and CGF have been reported. PRF and A-PRF released, in a constant way, a total amount of growth factors higher than PRP, which released most of the growth factors at the beginning of culture [4,8]. 

It has been reported that both A-PRF and CGF contain a higher amount of growth factors than PRP and PRF [5]. Moreover, analyzing the releases of some growth factors by CGF in an eight-day period, it has been shown that different growth factors had different release kinetics [9]. Plasma rich growth factors (PRGF) also contain multiple growth factors and cytokines; PRGF-modified collagen membranes allowed the kinetic release of these therapeutic molecules that enhanced bone regeneration and soft tissue healing [10]. However, to date, the growth factors released by CGF in a longer period have not yet been studied.

Some recent findings have opened interesting perspectives on the biotechnological use of CGF in the tissue regeneration field. The CGF-enhanced proliferation of three cultured cell lines (fibroblasts, endothelial cells, and osteoblasts) through the release of growth factors with specific kinetic accumulations, suggesting that a programmed release might support the regeneration process [9]. CGF alone is able to induce osteogenic differentiation of human bone marrow stem cells (BMSC) [11]. 

Several studies in vivo have stated improvements in tissue healing or regeneration in the presence of CGF [12]. It has also been reported that a better effect in bone formation occurs with CGF than with PRF in femur defects of adult dogs [13]. Moreover, a combination of CGF with stem cells or grafts determined better results than CGF alone [12]. 

Several authors have also shown that besides growth factors and platelets, the resident and circulating monocytes/macrophages and multipotent stem cells are important in the processes of tissue regeneration and differentiation [14,15]. 

Although a growing body of evidence suggests the existence of multipotent cells in peripheral blood [16,17], to date, the use of blood as an alternative source of autologous stem cells in regenerative medicine is limited by important questions: the predictability of successful isolation and ex vivo expansion by a standardized protocol. 

The aim of this work was the chemical, structural, and biological characterization of CGF to deepen the knowledge of this very promising biomaterial in the field of regenerative medicine. 

Here, we reported that CGF has a complex fibrin structure implicated in the release of growth factors, metabolites, and cells. These cells, which could regulate the production and sustain the release of the CGF growth factors, show stem features and are able to differentiate into osteoblasts. 

## 2. Results

### 2.1. Untargeted and Targeted GC/MS Metabolomic Analysis of CGF 

Gas chromatography coupled with mass spectrometry (GC-MS) is an ideal technique for identifying and quantifying metabolites of small molecules (<650 Da) [18]. Using an untargeted approach, the metabolomic profile of CGF was performed and compared with that of the PPP (platelet-poor plasma) fraction. PPP was the upper liquid fraction obtained after blood centrifugation, together with CGF. The results obtained did not lead to the identification of metabolites present exclusively in the CGF fraction. However, Table 1 shows the relevant results: it is possible to ascertain that CGF was enriched in L-glutamic and taurine. In fact, the amount of these metabolites was 0.56 mg/L and 3.82 mg/L respectively in the CGF fraction; whereas, it was 0.06 mg/L and 0.08 mg/L, respectively in the PPP fraction. 

### 2.2. Analysis of CGF Content and Release of Growth Factors and Matrix Metalloproteinases 

In the present study, we determined the presence of bioactive molecules in CGF by analyzing the initial quantities of growth factors and matrix metalloproteinases (MMPs) that were extracted by force just after CGF preparation. As reported in Table 2, we found that CGF contained growth factors such as VEGF, TGF-β1, and BMP-2, and their amounts were 792.8 ± 71.9 pg, 26.6 ± 3.1 ng, and 45.5 ± 5.7 pg, respectively. Moreover, we reported that CGF carried MMPs and the quantities of MMP-2 and MMP-9 were 321.1 ± 29.5 ng and 396.3 ± 34.3 ng, respectively (Table 2). 

In an attempt to mimic the natural release of bioactive molecules by CGF, we cultured the CGF, without any manipulation, in 2 mL of cell culture medium (L-DMEM) for a period of 0–28 days. At the indicated times (1, 3, 7, 14, 21, and 28 days), we collected an aliquot of CGF-conditioned medium (CGF-CM) for the determination of growth factors and MMPs. 

**Figure 1:**
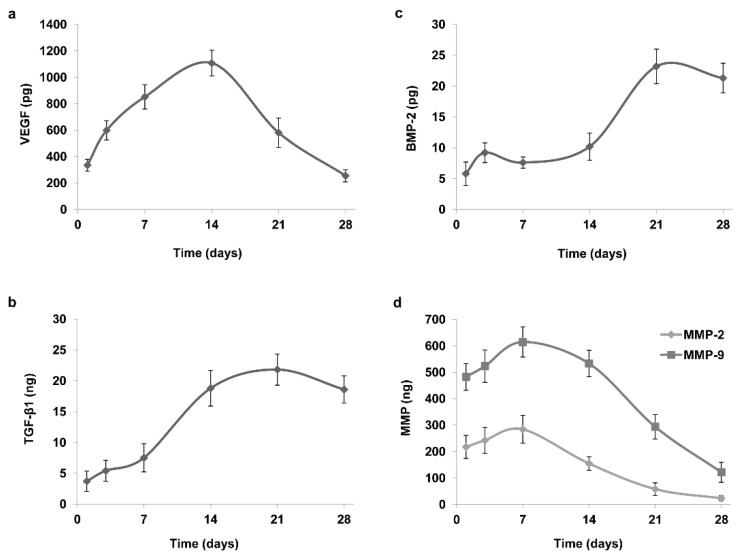
Growth factors and MMPs released by CGF. CGF clots were cultured in L-DMEM for a period of 0–28 days. At the appointed times (1, 3, 7, 14, 21, and 28 days), the conditioned medium was collected, and the growth factors (**a**) VEGF, (**b**) TGF-β1, and (**c**) BMP-2 and (**d**) the matrix metalloproteinases MMP-9 and MMP-2 were quantified by ELISA. The results are expressed as the means ± SD of triplicate measurements from three independent experiments.

We found a significant amount of each molecule at each experimental time, 28 days after CGF preparation. As reported in Figure 1, each molecule exhibited its own specific release kinetics. VEGF appears to be released slowly up to 14 days after CGF preparation and was found to be present in the medium even after 28 days (Figure 1a). Indeed, VEGF quantity recorded after one day was approximately 335 pg, reaching the maximum level of about 1107.5 pg after 14 days, an even greater amount than that of VEGF extracted by CGF (792.8 pg). The VEGF amount gradually lowered up to 28 days, reaching values of 169.6 pg. TGF-β1 also appeared to be released slowly, peaked after 21 days, and remained high up to 28 days. TGF-β1 content was about 3.7 ng, 21.8 ng, and 18.6 ng after 1, 21, and 28 days, respectively (Figure 1b). The amount of BMP-2 was about 5.8 pg after one day, 23.2 pg after 21 days, and remained constant up to 28 days (Figure 1c). The release kinetics of MMP-2 and MMP-9 had the same trend, although the quantity of MMP-9 was higher than that of MMP-2 (Figure 1d). Indeed, the amount of MMP-2 and MMP-9 after one day was about 217.1 ng and 482.2 ng, respectively, reached the peak after seven days (284.2 ng for MMP-2 and 614.1 ng for MMP-9) and was significantly lowered after 28 days (22.7 ng for MMP-2 and 121.3 ng for MMP-9) (Figure 1d). Overall, the growth factors and MMPs released in CGF-CM reached quantities higher than the initial ones extracted from CGF.

### 2.3. CGF: Fibrin and Cellular Components 

**Figure 2:**
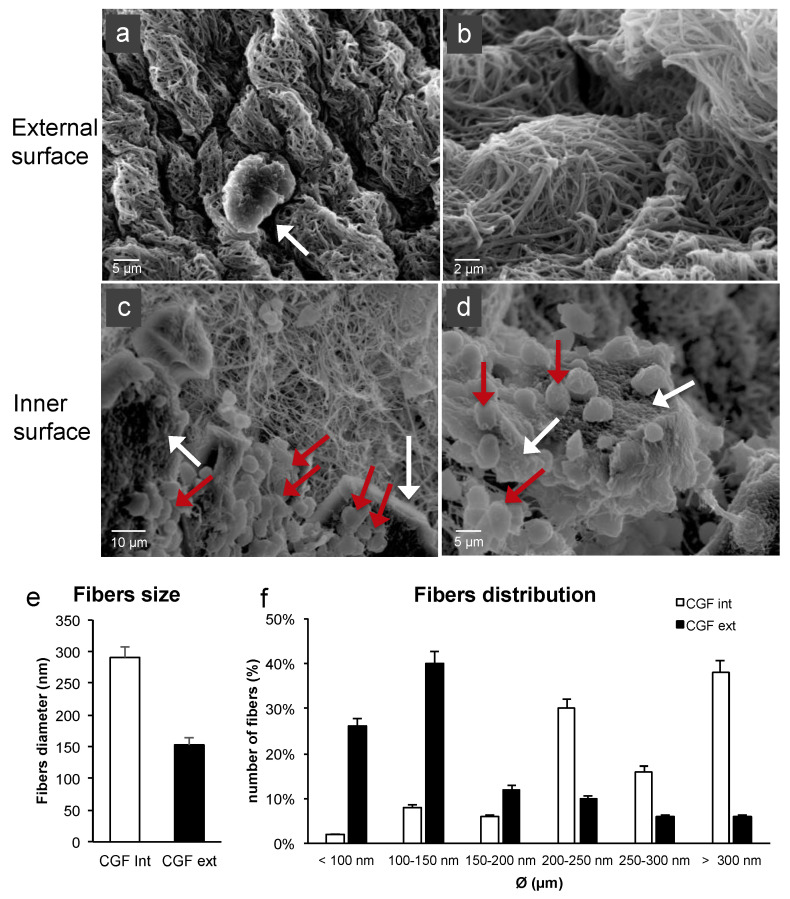
SEM images of fresh CGF. (**a**) The external surface of CGF was characterized by few activated platelets (white arrow) within the fibrin matrix. (**b**) Fibrin network appeared densely packed. (**c**,**d**) The inner surface of CGF showed a large population of activated platelets (white arrows) and white blood cells (red arrows). (**e**,**f**) Average diameters and size distribution of fibrin fibers were calculated using ImageJ software. The results were expressed as the means ± standard deviation (SD) of 50 measurements from each acquired sample.

To evaluate the features of the fibrin network and the cell content of CGF, the external and inner surfaces of its middle part were analyzed by SEM. The two surfaces showed different aspects. As shown in Figure 2a, on the CGF external surface, a dense fibrin network and few corpuscular elements, including activated platelets, were found (Figure 2b). The CGF inner surface presented high activated platelet zones and many cells (Figure 2c,d). 

The CGF fibers of the external surface seemed to be partially fused together. The fiber distribution analysis revealed an average diameter of 291 ± 16 nm and 153 ± 11 nm for the inner and external CGF surfaces, respectively (Figure 2e). Most of the fibers were included in the 100–150 nm range for the external surface and had a diameter larger than 300 nm for the inner surface. The distribution analysis highlighted that most of the fibers were included in the 100–500 nm range, similarly to the extracellular matrix (ECM) nanoarchitectures (Figure 2f). 

**Figure 3:**
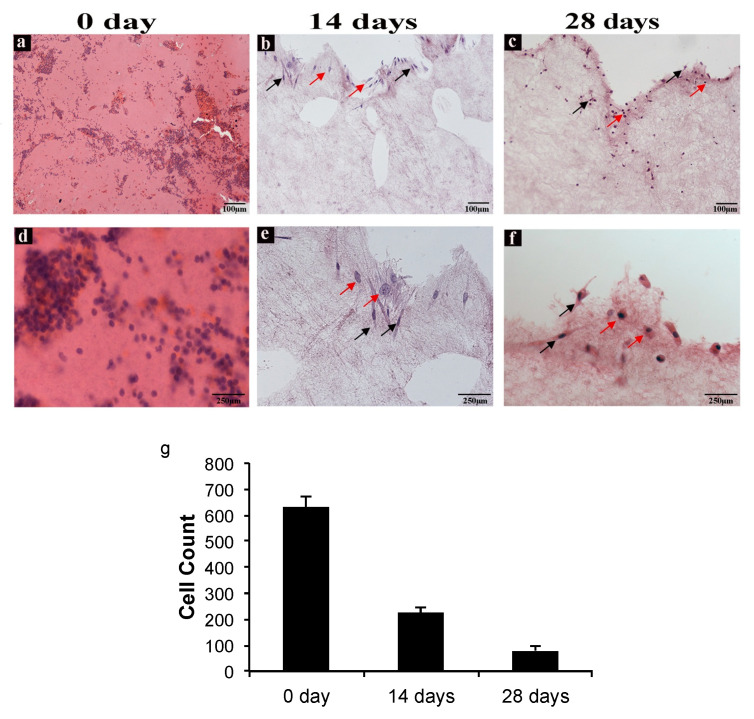
CGF hematoxylin-eosin staining. (**a**,**d**) Sections of CGF at time zero (0 day), (**b**,**e**) after 14 days, and (**c**,**f**) after 28 days of incubation in culture medium. The red arrows indicate the spherical cells, and the black arrows indicate the spindle-shaped cells. (**a**–**c**) Scale Bar: 100 μm, (**d**–**f**) 250 μm. (**g**) The number of cells in the CGF sections at 0, 14, and 28 days were calculated using ImageJ software. All values were expressed as mean ± SD (n = 3 per group, 3 replicates).

In order to evaluate cell distribution, density, and morphology in CGF, hematoxylin and eosin staining were carried out. Figure 3 shows images of CGF sections from three different experimental conditions: CGF prepared fresh (0 days), CGM cultured for 14 days, and CGM cultured for 28 days. 

A reduction of cell density in CGF sections at both 14 and 28 days with respect to that at 0 days was observed (Figure 3a–c) and confirmed through cell counts (Figure 3g). Moreover, at day zero, cell distribution was homogeneous all over the section, whereas at 14 and 28 days, the cells appeared isolated or were forming small groups, especially in the peripheral area of the sections. As shown in Figure 3, there are two morphologically different cell types: spindle-shaped and spherical. 

**Figure 4:**
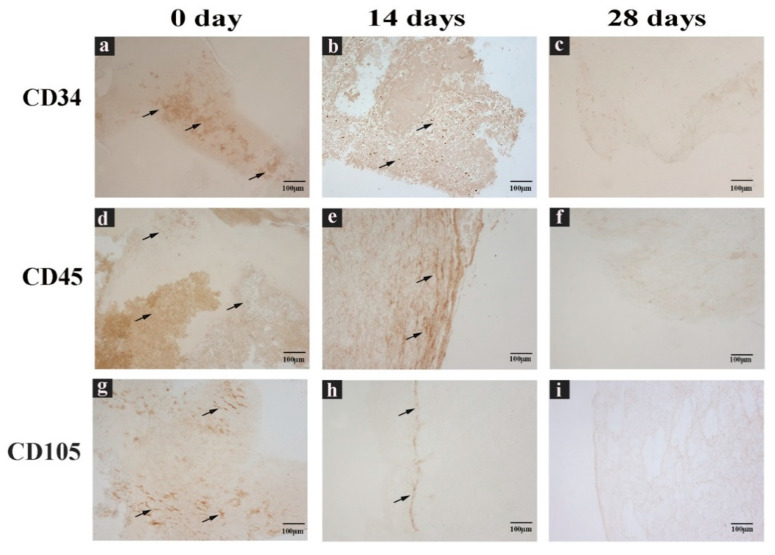
CGF sections labeled with immunohistochemical assay. (**a**,**d**,**g**) Sections of CGF at 0 days, (**b**,**e**,**h**) 14 days, and (**c**,**f**,**i**) 28 days of incubation in culture medium. Incubated with (**a**–**c**) anti CD34 antibody, (**d**–**f**) anti CD45 antibody, or (**g**–**i**) anti CD105 antibody. Black arrows indicate the positive immunolabel. Scale Bar: 100 μm.

Therefore, CGF cells were immunolabeled with anti-CD34, CD45, and CD105 antibodies directed against cell surface markers, to characterize their immunophenotype. Immunostaining showed CD34^+^, CD45^+^, and CD105^+^ cells (Figure 4). All stainings showed a reduction of immunoreactivity in CGF at 14 and 28 days compared to that at 0 days (Figure 4). 

### 2.4. CGF Cells Display Pluripotency and Stem Cell Markers

**Figure 5:**
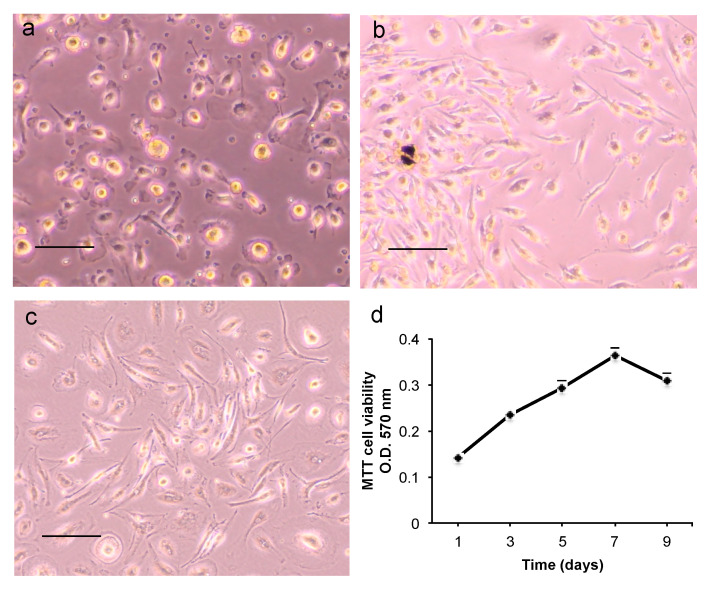
Morphology and cell proliferation of CGF primary cells. (**a**) Adherent cells released by the whole CGF at 14 days; (**b**) cells from CGF pieces after 14 days; (**c**) cells at third passage. Scale bar: 100 μm. (**d**) CGF primary cell viability was assessed by MTT assay on days 1, 3, 5, 7, 9 after cell seeding. Data represent mean ± SD of duplicate measurements from three independent experiments.

After 14 days in the culture medium, CGF released a mixture of cells (Figure 5a). In order to facilitate the release of the cells trapped in the CGF, the latter was chopped. Then the pieces were put into new culture plates where, after 7–10 days, a consistent population of adherent mononuclear cells was observed. Many cells had a spindle-shaped morphology, and few cells were spherical (Figure 5b). CGF adherent cells were able to proliferate, maintaining their own aspect across subsequent passages (Figure 5c,d). 

**Figure 6:**
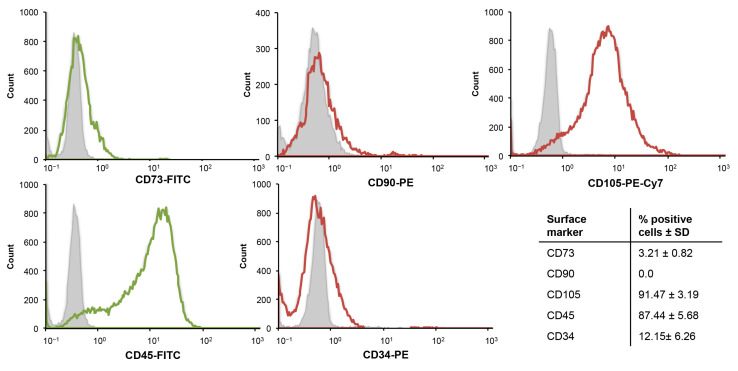
Flow cytometry analysis of mesenchymal and hematopoietic surface markers. Representative flow cytometry histogram of CGF cells. Grey histogram: isotype control; open histogram: signal for each specific antibody. Values in the table represent the percentage of positive cells for the dye as the mean ± SD.

To characterize CGF adherent cells, the expression of mesenchymal and hematopoietic stem cell markers was evaluated by flow cytometry. The analysis of hematopoietic markers showed that 90% of CGF cells expressed CD45. More than 90% expressed mesenchymal stem cell marker CD105, while other markers were not detected (CD90 and CD73) or were expressed at low levels (CD34) (Figure 6). 

**Figure 7:**
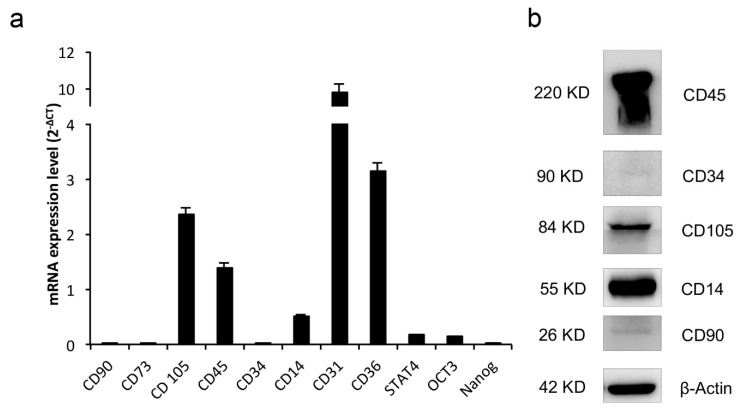
Gene expression of cell surface and pluripotent markers. (**a**) mRNA was quantified by real-time PCR in CGF primary cells. The comparative CT method (2^−ΔCT^ ± SD) was used to quantify the gene expression level. *Gapdh* was used as a housekeeping gene. The results are expressed as the mean ± SD of triplicate measurements from four independent experiments. (**b**) Expression of stem cell surface proteins. β-Actin was used as an internal loading control. The image is representative of three independent experiments.

The expression of cell surface markers and transcription factors required to maintain the pluripotency and self-renewal in stem cells (oct3/4 and Nanog) or to determine hema-topoietic development (Stat4) was analyzed by real-time PCR. CGF adherent cells showed high CD31, CD36, CD105, and CD45 mRNA levels; consistent mRNA levels of CD14, OCT-3, and STAT4 were also found, whereas low CD90, CD73, CD34, and Nanog mRNA levels were detected (Figure 7a). 

To further assess the expression of surface markers in cells released by CGF, a Western blotting analysis was carried out. In agreement with real-time PCR quantitation, CGF cells expressed high CD45, CD14, and CD105 protein levels. CD90 and CD34 protein levels were very weakly detectable (Figure 7b). 

### 2.5. Osteogenic Differentiation of CGF Primary Cells

**Figure 8:**
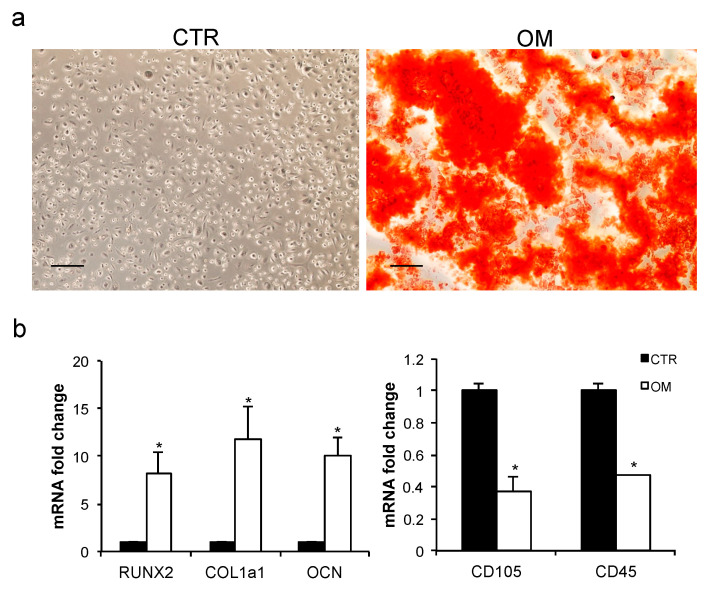
Osteogenic differentiation of CGF primary cells. (**a**) Alizarin Red staining after 21 days in culture medium (Control, CTR) or Osteogenic Medium (OM). Scale bar: 150 μm. (**b**) mRNA abundance of RUNX2, COL1a1, OCN in CGF primary cells cultured in culture medium or OM for 21 days. *Gapdh* was used as a housekeeping gene for normalization. The fold change in mRNA expression was relative to CTR. The results were expressed as the mean ± SD of triplicate measurements from three independent experiments (* *p* < 0.05 versus CTR).

To evaluate the capability of CGF primary cells to differentiate into osteoblasts, the matrix mineralization of these cells was analyzed by Alizarin red staining (ARS) experiments. After 21 days in osteogenic medium (OM), the CGF primary cells showed a very strong ARS staining when compared to the untreated primary cells kept in culture medium (CTR) (Figure 8a). To further assess the osteogenic potential of CGF primary cells, the mRNA abundance of RUNX2, the transcription factor key regulator of osteogenesis, of Collagen Type I Alpha 1 (COL1a1) and of Osteocalcin (OCN), extracellular matrix proteins used as osteogenic differentiation markers, was quantified after three weeks in osteogenic medium. RUNX2, COL1a1, and OCN mRNA levels markedly increased in cells incubated in OM with respect to CTR by about 7.3-, 10.7-, and 9.1-fold, respectively. This confirms, at a molecular level, the data obtained by ARS experiments (Figure 8b). After osteogenic induction, CGF primary cells also reduced the expression of stem cell surface marker CD105 and CD45 by about 0.6- and 0.5-fold, respectively. 

## 3. Discussion

In recent years CGF was widely studied as an autologous blood derivative able to promote tissue repair, vascularization, cell migration, and differentiation [11,19,20,21,22]. Tissue repair is a complex mechanism that takes place over four phases: inflammatory process, cell proliferation, differentiation, and ECM remodeling. The process involves cytokines, growth factors, and MMPs [15]. Despite a large literature on CGF use and applications in the regenerative medicine field [21,23], up to the present, no data are provided on the metabolomic profile of CGF, and very few studies investigated the kinetic release of CGF growth factors and MMPs over a long time and analyzed the CGF cellular component. The aim of this work was to characterize the CGF metabolites composition, the amount of growth factors and MMPs released by CGF over a period of 28 days, and to study in detail the CGF cellular components. 

GC/MS metabolomics analysis highlighted the high concentration of L-glutamic acid and taurine in CGF and the statistically different amount of the two analytes between the CGF and PPP fractions. These results are quite interesting considering the CGF application in the field of regenerative medicine. Indeed, it was demonstrated that ECM proteins and biomaterials, functionalized with amino acid sequences rich in glutamic acid, induced osteogenic differentiation, and mineralization of marrow stromal cells [24]. In fact, glutamic acid residues are known to act as a nucleation point for calcium phosphate mineralization [25]. Furthermore, taurine, a non-essential amino acid, has been shown to have positive effects on bone mass and influence bone metabolism [26]. Taurine was also shown to promote the differentiation of human MSC into osteoblasts and to upregulate the expression of osteoblast markers as osterix, Runx2, osteopontin, and alkaline phosphatase via ERK1/2 signaling [27]. In a recent study, we reported the ability of CGF to promote the osteoblast differentiation of BMSC [11]. This capacity could be due to the high levels of L-glutamic acid and taurine and to prolong release from CGF of some growth factors, as reported in the present study. In fact, the initial amount of some bioactive molecules extracted from CGF was analyzed soon after preparation, then their release from CGF was quantified over time. We found that CGF extract contained growth factors such as VEGF, TGF-β1 and BMP-2, and MMPs (such as MMP-2 and MMP-9), confirming previous studies [28,29,30]. Moreover, to mimic the natural release of soluble factors, we cultured CGF, without any manipulation, in cell culture medium, at different times, until 28 days. We found that growth factors and MMPs were gradually released over time up to 28 days from CGF preparation, following specific release kinetics. In particular, VEGF was released slowly up to 14 days, when it reached its maximum value and gradually decreased over time. Similar to VEGF, TGF-β1 and BMP-2 were also released slowly. They peaked at 21 days, and their values remained high up to 28 days. The matrix-degrading enzymes MMP-9 and MMP-2 were released faster than the growth factors and peaked after seven days, with MMP-9 more abundant than MMP-2, then gradually decreased over time. The present findings reported, for the first time, a continuous and prolonged release of multiple bioactive factors over time, suggesting that CGF is suitable in promoting the complex and long process of tissue regeneration. To the best of our knowledge, 28 days is the longest time analyzed for the release of factors from the CGF. Indeed, previous studies analyzed CGF growth factors release within shorter time intervals [9,31,32,33].

Two phases in the release of growth factors by CGF have been reported [34]: an immediate phase, which could be attributed to the instantaneous release from activated platelets during centrifugation or to simple diffusion; a late phase with accumulation peaks at 14 days, which could be explained by the release of growth factors after degradation of the fibrin structure and by the production of growth factors from the CGF resident cells [21,35,36]. 

Concordantly, we found that the growth factors and MMPs released in the conditioned medium from the CGF reached higher amounts than the initial ones extracted from the CGF, suggesting a role of CGF-resident cells in the synthesis and secretion of these factors. In particular, the amount of VEGF in the CGF-conditioned medium after a 14-day incubation was even greater than the amount of VEGF extracted from the CGF. These results agree with our previous study showing that CGF-derived cells expressed and released angiogenic factors, including VEGF [22].

Growth factors are considered essential elements in tissue regeneration; they play a critical role in regulating processes involved in wound healing and tissue repair, so their amounts and release kinetics, as we found, could be important to better assess the efficacy of CGF. 

Among the multiple growth factors released by CGF, VEGF is a crucial molecule in tissue repair and regeneration since it is implicated in angiogenesis, blood vessel growth from pre-existing vasculature and vasculogenesis, and de novo formation of blood vessels [37]. VEGF has been demonstrated to stimulate endothelial cell proliferation and promote angiogenesis by binding to a high-affinity receptor, and its signaling is considered a rate-limiting step in the initiation of angiogenesis [38]. However, due to the very short half-life of VEGF [39,40], low efficacy is achieved when administered as free proteins because high doses have a prohibitive cost and often cause undesirable effects [41]. Therefore, a sustained release system of VEGF is required to provide ideal therapeutic effects, which could be achieved by CGF application. 

In our experimental conditions, TGF-β1 was the most abundant growth factor contained in and released by CGF over time. TGF-β1 is a secreted protein that regulates many cellular functions, including the control of immune and stem cell growth, proliferation, differentiation, apoptosis, development, and tissue remodeling following injury [42,43]. Thus, the release of TGF-β1 is desirable in wound healing sites and particularly in the oral cavity, where several types of cells, like fibroblasts and osteoblasts, must be stimulated to proliferate. Temporal and spatial activation of TGF-β is involved in the recruitment of stem/progenitor cells and participation in the tissue regeneration/remodeling process. BMP-2, another important member of the TGF-β superfamily, plays a key role in the development of bone and cartilage. It is a highly potent growth factor able to promote the differentiation and maturation of osteoblasts [44]. We found that BMP-2 was the growth factor released by CGF in the lowest amounts. However, BMP-2 has been shown to be released from platelets, mainly at low pH [45], which is the common environment of wound healing sites [46]. Therefore, the use of CGF could improve the repair processes by locally stimulating the release of BMP-2 at the injury site. Moreover, we also found that CGF released the MMP-2 and MMP-9. MMPs are matrix-degrading enzymes implicated in many biological processes, including inflammation and cell migration during wound healing and tissue repair in coordination with several growth factors and cytokines [47].

The importance of the resident and circulating cells in the processes of tissue regeneration is well established [14,15]; therefore, besides growth factors and molecules contained in and released by CGF, we focused on the characterization of its cellular components. 

SEM observation did not reveal the presence of cells on the surface of CGF but showed a fibrin framework denser than inside of CGF, where large populations of activated platelets and cells were present. Immunohistochemistry analysis of CGF showed a very uniform distribution of nucleated cells entrapped in the fibrin network. The sections reacted positively to CD34, CD45, and CD105 immunolabelling. Indeed, the presence of different cell populations is known: hematopoietic stem cells, lymphocytes, monocytes, and fibroblast-like cells [1]. Our recent findings showed that when CGF, without manipulation, is released into the culture medium, cells are able to adhere to the plate and proliferate [22]. Here we show that the release of cells from CGF seemed to be rather slow, and most of the cells were found in the plate only after cutting CGF on the 14th day. This aspect could be correlated with hematoxylin-eosin staining data and with CGF fibrin network structure observed by SEM analysis: indeed, while at the initial stage CGF cell distribution was homogeneous all over the section, after two and four weeks, cells seemed to migrate from the center where fibrin network was less dense to the peripherical area of the sections, where fibrin appeared to be more densely intertwined. This scenario might explain either why cells were retained into CGF so long (up to 28 days) and the sustained release kinetics of CGF growth factors and MMPs. 

Di Liddo et al. recently reported that the leukocyte- and platelet-rich fibrin product called CPL-MB acts as an artificial stem niche containing autologous multipotent cells with defined stemness properties [48]. In our work, CGF primary cells showed fibroblast-like and spherical morphology; however, after few passages, cell populations appeared to be enriched in spindle-shaped cells and showed different surface markers with respect to cells resident in the CGF. Indeed, adherent cells expressed a high level of CD105 and CD45 surface markers; whereas, CD34 was scarcely detectable. Since we found that CGF primary cells exhibited monocyte markers, such as CD31, CD45, CD14, and CD36, [49,50] we assumed that they might be monocyte-derived cells. The primary CGF cells did not appear as mesenchymal stem cells derived from peripheral blood since they did not express CD73 and CD90 mesenchymal markers; however, they showed mesenchymal, hematopoietic, and endothelial stem cell features. Indeed, it has been demonstrated that monocyte-derived cells expressing CD105, CD45, and CD14 exhibit mesenchymal cell features and are able to differentiate into different cell lines [49]. 

In addition, CGF primary cells express genes that denote the molecular signature of stem cell pluripotency, including Oct3/4 and Nanog. The transcription factor Oct3/4 is thought to be indispensable for pluripotency in stem cells and is expressed in multipotent progenitor cells isolated from peripheral blood [17]. Nanog is a key factor in the self-renewing of embryonic stem cells, which remained pluripotent after multiple passages, but it has a heterogeneous expression mode; indeed, Nanog-negative cells show a higher propensity for differentiation [51]. Our results show low Nanog mRNA levels. We also reported high STAT4 mRNA levels. STAT4 is a key transcription factor involved in promoting cell-mediated immunity, but its expression is not restricted to lymphoid cells. Activated monocytes expressed STAT4 in response to Interferon-alfa [52], a cytokine that downregulates osteoblastogenesis [53], though increases the formation of calcific nodules under osteogenic conditions in human aortic valve interstitial cells [54]. 

Finally, to better characterize the use of CGF in the field of regenerative medicine, since CGF primary cells seem to display several pluripotency markers, the ability of these cells to differentiate into osteoblasts was tested. Interestingly, we found that CGF primary cells, kept three weeks in osteogenic medium, were able to differentiate into osteoblasts as demonstrated by the formation of mineralized nodules, the expression of the osteogenic markers RUNX2, COL1a1, and OCN, and the loss of stem cell markers [11]. These results suggest that CGF could also represent a source of cells with stem features, thus expanding its potential applications. 

Recently, we demonstrated the ability of CGF to promote the osteogenic differentiation of stem cells [11]. Furthermore, we showed that CGF releases endothelial progenitor cells, which contribute to neo-angiogenesis and to the formation of endothelial tubular structures [22]. 

Here we reported that CGF has a complex inner structure capable of influencing the release of growth factors, metabolites, and cells. These cells, which could regulate the production and release of the CGF growth factors, show stem features and are able to differentiate into osteoblasts, producing a mineralized matrix. These data, taken together, highlight interesting new perspectives for the use of CGF in tissue regeneration and in regenerative medicine.

## 4. Materials and Methods

### 4.1. Preparation of CGF 

Blood samples of 8 mL were taken via venipuncture from ten (seven male and three female) non-smokers in generally good health. Informed consent was obtained from the donors included in this study. Tubes of blood were processed by a device (Medifuge MF200; Silfradent srl, Forlì, Italy) to obtain CGF; each blood sample was centrifuged for 13 min following the manufacturer’s instructions 2 min at 2700 rpm, 4 min at 2400 rpm, 4 min at 2700 rpm, and 3 min 3000 rpm. The centrifugation method used to obtain CGF created three fractions: PPP, the upper liquid fraction; CGF, the middle dense fraction and red blood cell, the lower fraction. The latter was excluded from the characterization analyzes. 

### 4.2. GC/MS Analysis

PPP and CGF metabolite extraction and analyses were carried out as previously reported [55]. Briefly, about 30 mg of PPP or CGF were extracted with 1 mL nitrogen-degassed and cooled solvent consisting of a ternary mixture of hydrophilic (water), lipophile (isopropanol), and medium polarity (acetonitrile) solvents in a ratio of 2:3:3 (*v*/*v*/*v*). An aliquot of the supernatant was taken after centrifugation at 14,000× *g* for 2 min, dried, and resuspended in an equal volume of nitrogen-degassed 50:50 (*v*/*v*) acetonitrile/water at room temperature and centrifuged at 14,000× *g* for 2 min. The supernatant, after transfer into a clean vial, was added to the internal reference standards consisting of a homologous series of n-alkanes (C8-C40) and 10 mL of 10 mg/L solutions of norleucine and dried. After protection of carbonyl moieties by methoximation with 10 µL of a 20 mg/mL solution of methoxyamine hydrochloride in pyridine at RT for 90 min, derivatization was carried out with 50 µL of N-methyl N-tert-butyl-dimethylsilyl-trifluoroacetamide (MTBSTFA) at 70 °C for 1 h. Blanks and external reference QC mixtures were prepared in the same manner.

One microliter was injected in a pulsed split-less mode for 1 min at 7.2 psi into a GC/MS system consisting of a 7683 autosampler, a 6890N GC, and 5973 inert single quadrupole mass spectrometer detector (all Agilent Technologies, Milan, Italy). The injection temperature was 250 °C, the interface was set to 280 °C, and the ion source was at 230 °C. Metabolite separation was performed on a DB-1HT column (30 m, id 0.32 mm, film thickness 0.1 μm) using a Helium flow of 1 mL/min. After 2 min at 50 °C, the oven temperature was increased by 10 °C min^−1^ up to 350 °C, then 15 min isocratic for an overall chromatographic run of 47 min. Mass spectra were recorded from 50 to 600 *m*/*z* at 0.5 s/scan. ChemStation (version D01.01, Agilent Technologies, Santa Clara, CA, USA) and AMDIS (automated mass devolution and spectral identification system, version 2.65, NIST, Gaithersburg, MD, USA).

Software were used for the processing of the acquired data. Mass spectra of all detected compounds were compared with spectra in the NIST library, an in-house mass spectra library database, or the Golm Metabolome Database (http://gmd.mpimp-golm.mpg.de/, accessed on 15 June 2021). 

### 4.3. Growth Factors and MMPs Content and Release 

After preparation, each CGF clot was washed with phosphate buffer saline (PBS), to remove excess serum and processed following experimental protocols, as detailed below. 

In the groups for immediate extraction by force, each CGF clot was promptly frozen at –80 °C, then cut into small pieces and homogenized in 1 mL sterile cell culture medium (low glucose-Dulbecco’s modified eagle medium, L-DMEM) using a potter. Then, a final centrifugation (1500 rpm for 10 min) was performed to remove residual particulates. About 1 mL of solution was collected, aliquoted, and stored at −80 °C until analysis. 

In the cultured CGF groups, each CGF clot was placed in a 12-well plate (one in each well) with the addition of 2 mL of cell culture medium (L-DMEM), supplemented with 100 U/mL penicillin/streptomycin and without fetal bovine serum (FBS), and incubated at 37 °C in a humidified atmosphere with 5% CO_2_ for a period of 0–28 days. After each incubation period (1, 3, 7, 14, 21, and 28 days), 400 µL of CGF-conditioned medium (CGF-CM) was collected and replaced with 400 µL of fresh culture medium. Then CGF-CM was centrifuged at 1500 rpm for 10 min at room temperature, and the supernatant was aliquoted and stored at −80 °C until analysis. 

The growth factors VEGF, TGF-β1, and BMP-2, and the matrix metalloproteinases MMP-9 and MMP-2 released in CGF-CM, were quantified using commercial human ELISA kits, according to the manufacturer’s instructions. 

The total quantity of growth factors present in the medium recovered at all time points was checked and reported as a mean value at each time point. 

### 4.4. SEM Analysis

The CGF was fixed in 4% (*w*/*v*) paraformaldehyde (PFA) in PBS for 2 h (room temperature), followed by two PBS washings, and final storage in 0.05% (*w*/*v*) sodium azide in PBS. After fixation, the CGF was rinsed two times with PBS, dehydrated in scalar ethanol/water solutions (15%, 25%, 50%, 70%, 90%, and 100% ethanol, 10 min each), and then freeze-dried. To observe the inner surface, CGF was cut in the middle with a scalpel, along the transverse plane, and coated with a 7 nm layer of gold and examined under scanning electron microscopy (SEM EVO^®^ 40, Carl Zeiss AG, Oberkochen, Germany), in variable pressure mode with an accelerating voltage of 20 kV. The sample was placed on the SEM sample holder using double-sided adhesive tape and was observed without any further manipulation at a lower and higher magnification (2 kX and 10 kX). SEM micrographs were then analyzed by ImageJ 1.50c software (NIH, https://rsb.info.nih.gov/ij, accessed on 18 March 2021) to evaluate the average fiber diameter and size distribution in the fibrin matrix (50 measurements for each acquired sample). The diameters are reported as the mean ± standard deviation. 

### 4.5. Immunohistochemistry and Cell Count

CGF samples were incubated in L-DMEM supplemented with 10% FBS, 100 IU/mL penicillin/streptomycin, and 100 IU/mL L-glutamine for 28 days. Three different times were considered: 0 days, 14 days, and 28 days of incubation. The medium was replaced at a rate of 50% once/week. Samples were fixed by immersion in 4% paraformaldehyde in PBS pH 7.4 at room temperature, followed by dehydration, standard paraffin embedding procedure, and sectioning with a rotating microtome (Leica, Milan, Italy) to obtain 10 μm sections. Sections were stained by either standard Hematoxylin and Eosin or immunohistochemistry procedure. In the latter, samples were incubated either with a mouse primary monoclonal antibody (mAb) anti-CD34 (Santa Cruz, Heidelberg, Germany) overnight, a rat mAb anti-CD105 (Santa Cruz) overnight (4 °C), or a mouse primary monoclonal antibody (mAb) anti-CD45 (Santa Cruz) overnight. Then, they were properly incubated for 1 h with a biotinylated anti-mouse Ab (Dako, Milan, Italy) and with a biotinylated anti-rat secondary Ab (Millipore, Milan, Italy). To detect the formation of the antigen-Ab complex, sections were incubated for 1 h with extravidin peroxidase (Sigma-aldrich, Milan, Italy), and color development was obtained with 3,3-diaminobenzidine (Sigma-aldrich).

Sections, for each time, stained by standard hematoxylin and eosin method were analyzed by ImageJ software to obtain cell counts. 

### 4.6. Isolation and Culture of Primary Cell Populations from CGF

The CGF was washed twice with PBS; then, it was placed into the cell dishes, covered with L-DMEM medium supplemented with 10% FBS, 100 IU/mL penicillin/streptomycin, 2 mM L-glutamine, and incubated at 37 °C with 5% CO_2_. The medium was replaced at a rate of 50% once/week. After 14 days, CGF was chopped into small pieces, which were plated in 35 mm cell dishes and cultured with L-DMEM. When the cells migrated from CGF and adhered to cell dishes, the CGF pieces were discarded, and the culture medium was changed twice a week. At 80% confluence, primary cells were detached using 0.02% EDTA/0.25% trypsin solution and seeded at a density of 5 × 10^3^ cells/cm^2^. All experiments were conducted on primary cells between the third and fifth passages. 

### 4.7. Cell Surface Marker Analysis by Flow Cytometry

Primary cells were analyzed after three passages by flow cytometry for the expression of the surface markers. Cells were harvested and incubated for 30 min with antibodies (eBioscience) against hematopoietic markers (CD45-FITC, CD34-PE) and mesenchymal stem cell markers (CD105-CyPE, CD73-FITC, CD90-PE). As a negative control isotype, antibodies conjugated with FITC and PE were used. The cytofluorimetric analysis was performed with CyFlow space (Partec-sismex), and the data were analyzed using FloMax software. 

### 4.8. Proliferation Assay

Cell proliferation was determined using the 3-(4,5-dimethylthiazolyl-2)-2,5-diphenyltetrazolium bromide (MTT) assay at different time points. MTT is a commonly used method to evaluate the presence of metabolically viable cells, based on the ability of viable cells to convert MTT, a soluble tetrazolium salt, into an insoluble formazan precipitate which is quantitated spectrophotometrically. Briefly, the cells were seeded at 1.5 × 10^4^ cells/mL into a 24-well plate and 0.5 mL of culture medium containing 50 µL of MTT stock solution, 5 mg/mL in phosphate-buffered saline (PBS) solution, were then added to each well. After a 2 h incubation, the MTT solution was removed, and 0.5 mL of 0.01 N HCl in isopropyl alcohol was added to solubilize formazan crystals. Absorbance was measured at 570 nm by a spectrophotometer. 

### 4.9. Western-Blot Analysis

To obtain whole protein cell extracts for Western-blot analysis, cells released by CGF and cultured into cell dishes were scraped in the following buffer: 20 mM Tris–HCl (pH 8.0), 420 mM NaCl, 2 mM EDTA, 2 mM Na_3_VO_4_, and 1% (*v*/*v*) Nonidet P-40, supplemented with a cocktail of protease inhibitors. Cells were then passed several times through a 20-gauge syringe and centrifuged at 16,000× *g* for 20 min at 4 °C. Proteins in homogenate were determined using the Bio-Rad protein assay kit. Lyophilized bovine serum albumin (BSA) was used as a standard. Total cell proteins were dissolved in sodium dodecyl sulfate (SDS) sample buffer and separated on 10% (*w*/*v*) SDS gels. Separated proteins were then transferred electrophoretically onto a nitrocellulose membrane (Pall, East Hills, NY, USA). Equal protein loading was confirmed by Ponceau S staining. The filter was blocked with 5% (*w*/*v*) non-fat dried milk in buffered saline. Blots were incubated with specific primary antibodies, and the immune complexes were detected using appropriate peroxidase-conjugated secondary antibodies and enhanced chemiluminescent detection reagent (Amersham International, Corston Bath, UK). Densitometric analysis was carried out on the Western blots by using ChemiDoc MP Image System (BioRad, Segrate (Mi), Italy). 

### 4.10. Real-Time PCR 

Total RNA was extracted from cells grown in a 35 mm ∅ culture dish using the Trizol (Sigma, Merck Life Science S.r.l., Milan, Italy) following the manufacturer’s protocol. The reverse transcriptase reaction (20 µL) was carried out using 1 μg of total RNA, random primers, and MultiScribe^®^ Reverse Transcriptase (Applied Biosystem, Monza, Italy) according to the manufacturer’s protocol. Quantitative gene expression analysis was performed in a CFX Connect Real-time System (BioRad, Segrate (Mi), Italy) using SYBR Green technology (FluoCycle-Euroclone, Milan, Italy). Primers used in real-time PCR are reported in Table 3. The efficiency of each primer was tested using a standard curve in duplicate. The quantifications were performed using the ΔΔCT or ΔCT method, and the *Gapdh* gene was used as an internal control for normalization. The specificity of the PCR products was confirmed by the melting curve analysis.

### 4.11. Osteogenic Differentiation Protocol

Primary CGF cells were cultured in L-DMEM supplemented with 10% FBS, 100 IU/mL penicillin/streptomycin, 2 mM L-glutamine, and incubated at 37 °C with 5% CO_2_. To induce osteogenic differentiation, CGF primary cells were cultured in L-DMEM with 10% FBS, 100 IU/mL penicillin/streptomycin, 2 mM L-glutamine, 10 mM β-glycerophosphate, 100 nM dexamethasone, 100 μM ascorbic acid 2-phosphate, for 21 days. The medium was replaced at a rate of 50% every 3 days.

### 4.12. Alizarin Red Staining

Alizarin red S stain (Sigma) solution was prepared as described in [11]. Briefly, Alizarin red S stain 2% solution in distilled water was adjusted to pH 4.2 by adding ammonium hydroxide drop-by-drop while stirring, using an electrode pH meter. The solution was then filtered through a 0.45 μm microfilter (Millipore Corporation, Bedford, MA, USA) and kept in an amber bottle. This solution was refiltered through a 0.22 μm microfilter immediately before use. The primary CGF cells, 4.5 × 10^4^ viable cells/mL, were seeded in a 12-well culture plate. After 24 h, the culture medium was refreshed. Cells were grown in culture medium, or osteogenic medium (L-DMEM with 10% FBS, 100 IU/mL penicillin/streptomycin, 2 mM L-glutamine, 10 mM β-glycerophosphate, 100 nM dexamethasone, 100 μM ascorbic acid 2-phosphate), for 21 days. ARS of primary CGF cells was performed at 21 days to detect osteoblast calcification. Cells were washed twice with PBS, fixed in 4% (*v*/*v*) paraformaldehyde in PBS for 15 min, washed with distilled water three times, and then stained by Alizarin Red S staining solution. After being rinsed twice with distilled water, the cells were photographed.

### 4.13. Statistical Analysis

Values were expressed as mean ± SD for the indicated number of experiments. Differences between the two groups were settled by unpaired Student’s *t*-tests. In all comparisons, *p* < 0.05 was considered statistically significant. Cell count statistical analysis was performed using Statgraphics Centurion (Statpoint Technologies Inc., Warrenton, VA, USA); the statistical significance of the differences was evaluated by ANOVA.

## Figures and Tables

**Table 1:** Metabolites with their respective concentrations identified in the CGF and PPP fractions. Concentration of metabolites expressed as mg/L.

Compound	R.T. (min)	Qion (m/z)	Concentration (mg/L)	
			CGF	PPP
L-Alanine	10.614	158	1.44 ± 0.03	1.51 ± 0.03
Glycine	10.843	218	0.83 ± 0.06	0.62 ± 0.04
L-Valine	11.903	186	1.08 ± 0.01	1.24 ± 0.01
L-Leucine	12.371	200	0.49 ± 0.03	0.47 ± 0.03
Isoleucine	12.672	200	0.23 ± 0.02	0.21 ± 0.02
L-Proline	14.564	184	0.01 ± 0.00	0.01 ± 0.00
L-Threonine	15.528	404	0.47 ± 0.02	0.62 ± 0.02
L-Phenylalanine	15.901	234	0.22 ± 0.00	0.22 ± 0.00
L-Aspartic	16.485	418	0.09 ± 0.01	0.05± 0.01
L-Glutamic	17.640	432	0.56 * ± 0.04	0.06 ± 0.00
L-Histidine	19.426	196	0.13 ± 0.01	0.13 ± 0.01
L-Tyrosine	19.860	466	0.21 ± 0.02	0.21 ± 0.02
Taurine	14.154	296	3.82 * ± 0.11	0.08 ± 0.02
FA18	19.035	341	47.9 ± 5.08	45.84 ± 4.87
FA16	17.743	313	63.95 ± 0.53	63.27 ± 0.57
FA14	16.065	285	3.57 ± 0.07	3.37 ± 0.06
FA12	14.201	257	1.99 ± 0.05	1.61 ± 0.04
FA10	12.187	229	1.3 ± 0.04	1.35 ± 0.03
FA8	10.039	201	2.29 ± 0.03	2.17 ± 0.04

Data represent the means of three independent experiments measured twice (* *p* < 0.01). R.T.: retention time; Qion: quantification ion; RSD: relative standard deviation.

**Table 2:** Growth factors and MMPs extracted from CGF.

Molecules	Quantity
VEGF	792.8 ± 71.9 pg
TGF-β1	26.6 ± 3.1 ng
BMP-2	45.5 ± 5.7 pg
MMP-2	321.1 ± 29.5 ng
MMP-9	396.3 ± 34.3 ng

The bioactive molecules were analyzed by ELISA, and the results are expressed as the means ± SD of triplicate measurements from three independent experiments.

**Table 3:** Oligonucleotides used for real-time PCR analysis.

Gene Name	Accession Number	Sequences (5′–3′)	pb
Thy1 (CD90)	NM_006288.5	F: ccactctggccattccc R: gagcaggagcagcagcag	124
CD73	BC015940.1	F: agcttacgattttgcacacc R: cggatctgctgaaccttgg	133
Endoglin (CD105)	NM_001278138.1	F: gccagcattgtctcacttca R: atgcgcaacaagctctttct	180
CD34	M81104.1	F: caatgaggccacaacaaaca R: gtgactggacagaagagttt	101
PTPRC (CD45)	NM_080921.3	F: atgaccatgtatttgtggctta R: tgggggaaggtgttgggc	97
CD31	NM_000442.5	F: atgatgcccagtttgaggtc R: acgtcttcagtggggttgtc	172
CD36	NM_001001548.3	F: agatgcagcctcatttccac R: gccttggatggaagaacaaa	115
CD14	NM_000591.4	F: acctaaagataaccggcacc R: ttgggcaatgctcagtacct	163
STAT4	NM_003151.3	F: aggaacggctgttgctaaag R: ttgtagtctcgcaggatgtc	193
Oct3	NM_002701.5	F: tattcagccaaacgaccatc R: gcaggaacaaattctccagg	219
Nanog	NM_024865.2	F: agatgcctcacacggagac R: tcttctgtttcttgaccggg	162
RunX2	NM_001278478.2	F:gacaaccgcaccatggtgg R: tctggtacctctccgaggg	160
Col1a1	NM_000088.3	F: agggaatgcctggtgaacg R: gagagccatcagcacctttg	90
Ocn	NM_199173.6	F: gctacctgtatcaatggct R: cgatgtggtcagccaactc	111
Gapdh	AJ005371.1	F: atggccttccgtgtccccac R: acgcctgcttcaccaccttc	245
